# Enhanced Recovery After Surgery: Are Benefits Demonstrated in International Studies Replicable in Pakistan?

**DOI:** 10.7759/cureus.19624

**Published:** 2021-11-16

**Authors:** Faiza H Soomro, Aneela Razzaq, Rameez Qaisar, Mehwish Ansar, Tehreem Kazmi

**Affiliations:** 1 General Surgery, The Dudley Group NHS Foundation Trust, Dudley, GBR; 2 Surgery, Shifa International Hospital Islamabad, Islamabad, PAK; 3 Surgery, Benazir Bhutto Hospital, Rawalpindi, PAK; 4 General Surgery, Pakistan Institute of Medical Sciences, Islamabad, PAK; 5 General Surgery, Shifa International Hospital Islamabad, Islamabad, PAK

**Keywords:** colorectal cancer, surgical site infection, length of hospital stay, enhanced recovery after surgery, colorectal surgery

## Abstract

Objectives

To determine the efficacy of enhanced recovery after surgery (ERAS) protocols in terms of frequency of surgical site infection (SSI) and length of hospital stay in patients undergoing colorectal surgeries for colorectal carcinoma.

Study design

Quasi-experimental study.

Setting/Duration of study

Department of Surgery, Shifa International Hospital, Islamabad, from May 7, 2019 to November 6, 2019.

Methodology

A total of 120 patients with colorectal carcinomas who fulfilled that sample selection criteria were studied. After randomization, patients were divided into two equal groups; one group received management under ERAS while the second group received conventional management. All patients were recorded for length of hospital stay and the development of SSIs. Data were analyzed using SPSS 26.0.

Results

The mean age was 42.34 ± 14.45 years, with a male majority, i.e., 72 (60%). The mean duration of in-patient stay was 3.45 ± 1.73 days with ERAS and 8.25 ± 1.58 days with conventional management (p < 0.001). A total of 28 (23.3%) SSIs developed, of which nine (7.5%) SSIs occurred with ERAS, while 19 (15.8%) occurred with traditional management (p = 0.031).

Conclusion

ERAS protocols have been demonstrated to be effective, cheap, and safe. There is a tangible reduction in length of hospital stay and incidence of SSIs which translates into reduced utilization of resources and financial costs. However, strict adherence to the protocol may be necessary to obtain the aforementioned benefits, which may be difficult to do in the face of professional, institutional, and personal inertia. Intensive efforts are required to make these protocols more convenient and attractive to implement, so as to facilitate conversion to this management approach.

## Introduction

Colorectal cancer is the third leading cause of cancer mortality globally, accounting for 10.2% of all cancers, with an incidence of 1.85 million new cases per annum [1]. Approximately 90-95% of the cases are sporadic, usually associated with risk factors like advanced age, male gender, high fat intake, obesity, alcohol, high red meat intake, smoking, and a lack of physical exercise [2,3].

Evidence-based management is the cornerstone of modern healthcare therapeutics, and the treatment of colorectal cancer is no exception. Despite lack of evidence, practices such as preoperative bowel preparation in colorectal surgery, routine use of nasogastric tubes, and nil by mouth instructions until bowel sounds are audible postoperatively are still widely practiced [4,5]. Factors such as pain, prolonged immobilization, and post-surgery ileus can lead to lengthened hospital stays and higher complication rates after major colorectal surgery [6].

Colorectal surgery is associated with a high rate of surgical site infections (SSIs), reported at rates of 5%-27%, with an exceptionally high incidence in patients with stoma formation, increased operative time, higher American Society of Anaesthesiologists' (ASA) grades, contaminated wounds, smoking, and the presence of immunosuppressive states [7]. Interventions that are used to mitigate the development of SSIs include perioperative antibiotics, hair removal, antiseptic skin preparation, maintenance of normothermia, and strict glycaemic control, with varying degrees of success [7].

Enhanced recovery after surgery (ERAS) protocols are evidence-based measures aimed at the standardization of perioperative management of the patient to optimize surgical outcomes [7]. These protocols target patients’ surgical stress response, postoperative morbidity, mortality, length of hospital stay, patients' perceptions of the surgical experience, and health care costs [8]. Specific interventions include a focus on anaesthesia, analgesia, reduction of surgical stress, goal-directed fluid therapy, the prevention of nausea and ileus, thromboembolic prophylaxis, minimally invasive techniques, nutrition, and early mobilization, ultimately resulting in shortened hospital stay [8].

Miller TE et al. in 2014 reported a decreased median length of hospital stay of five days with ERAS compared to seven days with standard management (p < 0.001), however, there was no difference in the occurrence of SSIs, 28.8% versus 37.3%, respectively, (p = 0.16) [9]. Ripollés-Melchor J et al. in 2018 reported that the mean length of hospital stay was 11 ± 10 days with ERAS versus 13 ± 17 days with traditional care (p = 0.034). However, in contrast to Miller TE et al., the frequency of deep SSIs was 3.4% in the ERAS group versus a frequency of 11.6% in the conventional care group (p < 0.001) [10].

Colorectal carcinoma is alarmingly common, resulting in significant morbidity and mortality and a major resource burden. Surgery has a potentially curative role, but most patients have poor functional status and poorly tolerate this management modality. Prolonged hospital stays cause further deterioration. The goal of this study was to compare the differences in patient outcomes with and without ERAS in terms of SSI rate and length of hospital stay, which would help to provide evidence in favor of or against practices currently in vogue.

## Materials and methods

**Figure 1:**
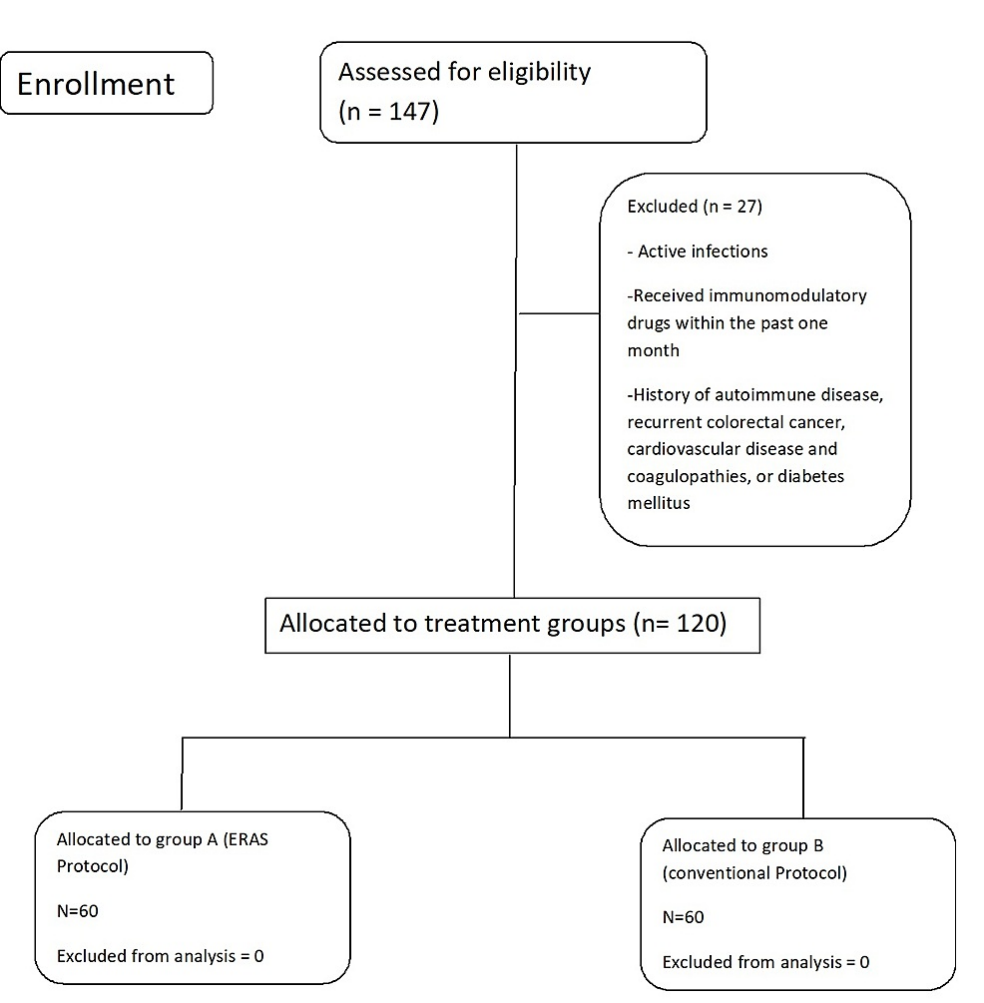
Consort flowsheet showing patient allocation to study groups.

We conducted a quasi-experimental study from May 2019 to November 2019 in the Department of Surgery, Shifa International Hospital, Islamabad, on 120 patients diagnosed with colorectal carcinoma after receiving informed consent. Patients were selected via consecutive non-probability sampling after receiving the appropriate ethical clearance. The WHO sample size calculator was used to calculate the sample size keeping a level of significance (ɑ) of 5×10-9, power of the test (1-β) of 100%, population SD of 1.735, [11] test value of the population mean of 8.5 [11] and an anticipated population mean of 3.8 [11]. Patients between the ages of 20 and 70 years undergoing elective surgery for colorectal cancers were included in the study. The types of surgeries included were open/laparoscopic right or left colectomies; subtotal colectomies, anterior resections, high anterior resections, and abdominoperineal resections. Those patients who had active infections or received immunomodulatory drugs within one month or had a history of autoimmune disease, recurrent colorectal cancer, cardiovascular disease, coagulopathies, or diabetes mellitus were excluded. All patients then underwent a thorough history and detailed physical examination. In Group A, patients received their management under the ERAS protocols [12]. While in Group B patients, colorectal surgery was performed using conventional management as per institutional protocols. The same surgical team conducted all surgeries; each surgeon had a minimum of five years post-fellowship experience in colorectal surgery (Figure 1).

The ERAS protocols applied include a carbohydrate-loading drink two hours before surgery; shorter fasting time (at least six hours for solids and two hours for liquids); early enteral feeding; early removal of gravity drains, nasogastric tube and urinary catheters; analgesia avoiding opioids and early mobilization and thromboprophylaxis. The conventional protocols as per the institutional policy include delayed resumption of enteral feeding; prolonged fasting time (minimum 8 hours); keeping of abdominal drain until fluid < 50 ml; adequate analgesia including opioids; preoperative bowel preparation (soft diet from 3 days prior to surgery and only liquid diet 16 hours prior to surgery).

The operative time from induction of anesthesia till placement of final closing suture was documented for each patient. All patients were monitored for the development of SSIs until discharge, at two weeks and four weeks post-surgery. The primary surgeon examined each patient themselves, classifying the SSI into superficial, deep, and organ space infections. The hospital stay was recorded in terms of the number of days stayed in the hospital from the time of surgery till discharge. The site of tumor and operative procedure performed, as well as total operation time, were documented.

Data were analyzed using SPSS version 26.0. Mean and SD were calculated for quantitative variables like age, BMI, operation time, and hospital stay. Qualitative variables like gender, location of the tumor, operative procedure, and SSIs were recorded in terms of frequency and percentage. The Chi-square test was applied to all variables for comparison with the development of SSIs between groups. An independent sample T-test was used to compare hospital stay between the two groups. The p-value of ≤0.05 was taken as significant.

## Results

We studied 120 patients divided into two equal groups. The mean age of the patients in our sample was 42.34 ± 14.45 years, with the majority of these patients being male, i.e., 72 (60%). The mean BMI in our patients was 27.76 ± 3.31 kg/m2. A total of 66 (55%) patients suffered from a disease involving the colon, while 54 (45%) had rectal disease. The presurgery characteristics are shown in Table 1. None of the variables demonstrated statistical differences across groups.

**Table 1:** Preoperative characteristics.

Variable	Group A	Group B	p-value
Age (years)	41.43 ± 14.28	43.25 ± 14.68	0.493
Gender			
Male	36 (30%)	36 (30%)	1.0
Female	24 (20%)	24 (20%)	
BMI (kg/m^2^)	27.85 ± 3.38	27.66 ± 3.25	0.763
Primary Tumour Site			
Colon	34 (28.3%)	32 (26.7%)	0.714
Rectum	26 (21.7%)	28 (23.3%)	

A total of 59 (49.2%) patients underwent open surgical procedures. In comparison, 61 (50.8%) received laparoscopic surgery. Two patients were converted from laparoscopic to open in group A, and three patients in Group B had to undergo conversion. A total of 13 (10.8%) patients were given a stoma, eight patients in group A and five patients in group B. The total mean operative time was 148.42 ± 19.32 minutes in our study, which was not statistically different across groups (p = 0.895). The mean hospital stay of the entire sample was 5.85 ± 2.92 days, the mean duration of stay was 3.45 ± 1.73 days with ERAS, and 8.25 ± 1.58 days with conventional management, p < 0.001. A total of 28 (23.3%) patients developed SSIs in our sample, of which nine (7.5%) SSIs occurred in group A, while 19 (15.8%) occurred in group B (p = 0.031). The postoperative surgical characteristics are displayed in Table 2. All patients who had SSIs were treated medically with antimicrobial therapy or open drainage of the wound in case of deep SSI. No patient needed any redo surgery for an organ space infection.

**Table 2:** Postoperative characteristics.

Variable	Group A	Group B	p-value
Type of Surgery			
Open	31 (25.8%)	28 (23.3%)	0.584
Laparoscopic	29 (24.2%)	32 (26.7%)	
Mean Operation Time (Minutes)	148.65 ± 19.26	148.19 ± 14.53	0.895
Mean Hospital Stay (Days)	3.45 ± 1.73	8.25 ± 1.58	<0.001
Surgical Site Infection			
Yes	9 (7.5%)	19 (15.8%)	0.031
No	51 (42.5%)	41 (34.2%)	

## Discussion

ERAS is a surgical guideline that prioritizes the reduction of surgical insult to the patient, rapid recovery, and an early return to physiological function, based on current evidence [13]. It has been demonstrated to reduce the incidence of adverse effects associated with surgery, thereby shortening recovery time, especially after colorectal surgery. Both these outcomes lead to an indirect reduction in the utilization of precious resources and costs [14]. Specific measures include adequate patient counseling, tailor-made diet plan, optimized analgesia, appropriate fluid and electrolyte supplementation, the use of minimally invasive surgical techniques, and encouraging early enteral intake and mobility [15].

Vlug MS et al. conducted a multi-center study on patients undergoing segmental colectomy using both open and laparoscopic procedures who received peri-operative care using ERAS protocols which were compared to standard care. The study found that the median total postoperative stay with laparoscopy/ERAS was 5 (interquartile range: 4-8) days, while it was slightly longer 6 (4.5-9.5) days with laparoscopic/standard management. This increased to 7 (5-11) days with open surgery coupled with ERAS, which became even more prolonged with the open surgery/standard care, i.e., 7 (6-13) days, (p < 0.001), figures that were also reflected in the post-surgery stay: 5 (4-7), 6 (4-8.5), 6 (4.5-10), and 7 (6-10.5) days, respectively, (p < 0.001). These findings were in keeping with our results, however, Vlug MS et al. concluded via regression analysis that it was laparoscopic surgery that was predictive of reduced hospital stay and morbidity and not the use of ERAS and that there was no difference between the groups with regard to perioperative morbidity (including SSI development), re-surgery and readmission rates, mortality, patient satisfaction, and total financial expenditure [16]. These findings were comparable to our findings in terms of hospital stay.

Greco M et al. in their meta-analysis concluded that ERAS in colorectal surgery resulted in a decreased all-cause morbidity (relative ratio [RR] = 0.60; 95 % CI: 0.46-0.76), however, the reduction in surgical complications such as SSIs was not reduced (RR = 0.76; 95 % CI: 0.54-1.08). A concrete advantage was seen in terms of shortened hospital stay (weight mean difference [WMD] = -2.28 days; 95 % CI: -3.09 to -1.47) [17].

Conversely, Zhuang CL et al. not only concluded that ERAS in colorectal surgery was associated with a statistically significant reduction to length of hospital stay (WMD = -2.44 days; 95% CI: -3.06 to -1.83 days; p < 0.00001), total length of admission (WMD = -2.39 days; 95% CI: -3.70 to -1.09 days; p = 0.0003), but was also associated with a reduction in the occurrence of complications such as SSIs (RR: 0.71; 95% CI: 0.58-0.86; p = 0.0006) [18]. Our data also showed a significant decrease in the rate of SSI in the ERAS group (p < 0.05).

However, the validity and utility of ERAS remain the subject of some debate, as a Cochrane review by Rawlinson A et al. concluded that while strict implementation of ERAS protocols was associated with decreased postoperative morbidity, reduction in hospital stays, and rapid return to function following colorectal surgery, the evidence clearly showed that it did not affect the rates of re-admissions or mortality [19]. Conclusions were echoed by yet another meta-analysis conducted by Currie AC et al. [20]. In our data presented, we have given the short-term follow-up and did not comment on readmission rates or mortality. We do plan to report another study with a larger cohort and an extended follow-up.

Another subject for debate is the rigorous requirements for the protocols to be successful, which invariably leads to a decrease in adherence. Pearsall EA et al. studied the attitudes of healthcare workers towards the implementation of ERAS and identified numerous problem areas, including a shortage of resources and manpower, inadequate communication, obstacles to teamwork, and a reluctance to change [21]. Ljungqvist O conducted a single-center study which showed that a 50-90% compliance rate with ERAS was associated with a reduction in total complication rate by 20%, as well a reduction in hospital stay by four days [22]. Pędziwiatr M et al. found that a minimum of 80% compliance with the complete ERAS protocol was mandatory before any benefit could be observed in terms of hospital stay and reduction of complications, and concluded that a minimum implementation time duration of six months, with at least 30 cases of surgery was required before this level of efficiency could be achieved [23]. Roulin D et al. found that compliance with ERAS, although high initially, decreases over time, especially in the postoperative period, with as high as 25% of cases having no medical basis for non-compliance [24].

Cavallaro P et al. conclude that the implementation of an ERAS protocol for colorectal resections necessitates a multi-disciplinary approach with dedication from both the surgery and anaesthesia departments [25]. The protocols need to be followed rigorously, in all phases of surgical management peri-operatively and require optimization of all aspects of patient care, including nutrition, fluid and electrolyte balance, early return to function, and adequate non-opioid analgesia. Adequate adherence translates into optimum outcomes, not a mean feat, as ERAS execution needs facilitation by dedicated support staff, prompt and open communication between all healthcare providers, and a handy checklist.

Ours was a single-center study, which was not blinded. The follow-up period was not long enough to assess long-term morbidity and mortality. Further research is required on this subject in Pakistan to assess the feasibility of implementing ERAS as a nationwide standard of care.

## Conclusions

ERAS protocols have been demonstrated to be effective, cheap, and safe. There is a tangible reduction in length of hospital stay and incidence of SSIs which translates into reduced utilization of resources and financial costs. However, strict adherence to the protocol may be necessary to obtain the aforementioned benefits, which may be difficult to do in the face of professional, institutional, and personal inertia. Intensive efforts are required to make these protocols more convenient and attractive to implement, so as to facilitate conversion to this management approach.
